# Effect of Central Motor and Neuromuscular Impairments on Front Crawl Body Roll Characteristics of Para Swimmers

**DOI:** 10.1186/s40798-025-00885-y

**Published:** 2025-08-01

**Authors:** Yu-Hsien Lee, Dawn Nicola O’Dowd, Luke Hogarth, Brendan Burkett, Carl Payton

**Affiliations:** 1 Department of Sport and Exercise Sciences, Manchester Metropolitan University Institute of Sport, Manchester, M15 6BH UK; 2 School of Health and Sport Sciences, University of the Sunshine Coast, Sippy Downs, QLD 4556 Australia

**Keywords:** Para swimming, Biomechanics, Classification, Motor impairment, Trunk rotation

## Abstract

**Background:**

Rotation of the trunk about its long axis or ‘body roll’ is essential for maximising front crawl swimming performance yet research on how physical impairment affects body roll is extremely limited. This study quantifies body roll kinematics in swimmers with and without central motor and neuromuscular impairments (CMNI). It was hypothesised that body roll kinematics differ between CMNI and non-disabled swimmers, are associated with sport class (level of impairment) and are influenced by upper and lower-limb functional levels.

**Methods:**

Three-dimensional motion analysis of 27 CMNI (sport classes 2–9) and 13 non-disabled competitive swimmers at 100–200 m race pace provided body roll kinematics, including shoulder and hip roll ranges and torso twist. Health conditions of the CMNI group were cerebral palsy (*n* = 12), spinal cord injury (*n* = 10) and neuromuscular disorders (*n* = 5). CMNI swimmers were divided into three upper-limb [mild (*n* = 9), moderate (*n* = 9), severe (*n* = 9)] and three lower-limb function subgroups [bilateral (*n* = 2), unilateral (*n* = 6), without kick (*n* = 19)] based on their Froude efficiency (a measure of how effectively upper limbs contribute to propulsion) and the number of lower limbs actively kicking during trials, respectively.

**Results:**

The CMNI group exhibited lower shoulder roll range (104 ± 11° vs. 88 ± 21°, *p* < 0.05) and torso twist (58 ± 13° vs. 48 ± 22°, *p* < 0.05) but greater hip roll range (62 ± 10° vs. 75 ± 29°, *p* < 0.05) than the non-disabled group. Statistical non-parametric mapping revealed less shoulder roll from 0 to 28%, less hip roll from 0 to 10%, greater hip roll from 91 to 100%, and less torso twist from 15 to 32% and from 75 to 81% of the cycle, in the CMNI than the non-disabled group (*p* < 0.05). CMNI body roll patterns varied widely, but discrete and continuous variables did not differ between upper-limb subgroups or between lower-limb subgroups.

**Conclusions:**

CMNI swimmers exhibit different body roll patterns to non-disabled swimmers. The study findings can be used to inform Para swimming coaches and improve the sport-specificity of land-based and water-based assessments currently used to classify CMNI swimmers.

## Key Points

- This is the first comprehensive study of body roll in Para swimmers. It establishes the effect of central motor and neuromuscular impairment (CMNI) on front crawl body roll kinematics and how this impacts swimming performance.
- Para swimmers with CMNI present considerable variation in their body roll strategies due to different levels of function in the upper limbs, trunk and lower limbs, either individually or in combination, reflecting unique self-organising adaptation to their individual organism constraints.
- Torso twist angles during non-disabled and CMNI front crawl swimming are notably lower than the threshold angle currently applied by World Para Swimming classifiers in their land-based test to define impaired trunk rotation. This indicates that the threshold may not be valid for classifying Para swimmers as it exceeds the amount of torso twist actually required during front crawl.

## Background

Front crawl swimming technique involves the use of asynchronous cyclic motions of the left and right upper limbs in the water, accompanied by shallow vertically-directed ‘flutter kicks’ from the lower limbs. These movements are synchronised with a rotation of the trunk about its longitudinal axis, commonly referred to as body roll. This trunk rotation is essential for maximising front crawl swimming performance [1]. Body roll involves rotation of the entire trunk combined with twisting of the trunk or ‘torso twist’ such that the hips and shoulders may achieve different amplitudes, possibly at different times in the upper limb cycle [2]. Body roll has been defined and quantified in a variety of ways [2–4]. A popular approach, when conducting kinematic analyses, is to quantify shoulder roll and hip roll by defining vectors through the two glenohumeral joints and hips joints, respectively [2, 3]. Alternatively, to help understand the kinetics of body roll, some researchers derive a total body roll angle through analysis of the whole body’s angular momentum [5, 6].

Body roll may facilitate the breathing action [7, 8], aid recovery of the upper limb [9], increase propulsion [10], decrease hydrodynamic drag [11] and reduce the risk of developing shoulder injuries [12]. Researchers have previously explored asymmetries in body roll and the association between body roll and swimming speed, stroke frequency, breathing action, skill level, and shoulder pain conditions, in non-disabled swimmers. These studies reported that: (i) male competitive swimmers, at sprint to 400 m pace, exhibit a total hip roll range of 37–57°, a total shoulder roll range of 97–111° and a total torso twist range of 61–78°, when the roll amplitudes from both sides of the body are summed [13], (ii) the ranges of shoulder and hip roll both decrease as swimming speed increases [5], (iii) body roll range is negatively associated with stroke frequency [14], (iv) swimmers roll their shoulders and hips more to the breathing side than the non-breathing side [8], (v) roll asymmetry does not seem to affect swimming performance [1], (vi) swimmers with unilateral shoulder pain roll their hips less compared to swimmers without [12], and (vii) swimmers use torso-twist to gain the benefits associated with shoulder roll without undue ‘waste’ of hydrodynamic force in non-propulsive directions [14].

Body roll has been analysed extensively in non-disabled front crawl swimmers due to its influence on performance. There are several mechanisms responsible for generating and controlling body roll: (i) hydrodynamic forces on the upper and lower limbs in medio-lateral and vertical (non-propulsive) directions create external torques which change the whole-body angular momentum about the longitudinal axis [2]; (ii) internal muscle torques generate reaction torques in the opposite direction to those driving limb movements [7]. These reaction torques mainly act to limit body roll amplitude rather than initiating the movement [2]; and (iii) the external torque produced by the buoyancy force. This occurs when the upper limb is above water during the recovery phase causing the whole-body centre of buoyancy to shift away from the whole-body centre of mass, creating a turning effect about the body’s longitudinal axis [15].

To date, only one experimental study has evaluated body roll in Para swimmers, a kinematic analysis of three unilateral arm amputees [3]. Thus, the vast majority of physical impairments eligible for Para swimming competition remain to be examined on this topic despite it being a key feature of the Technical Assessment (water test) component of the classification process where classifiers observe and score the athlete’s ability to ‘control their body….with particular attention to body streamline, body roll, trunk control and leg kick for balance…’ [16]. Classifiers currently have no benchmark data or detailed guidelines to aid their observation. Classification aims to minimise the impact an impairment has on the outcome of the event [17] by assessing the activity limitation caused by the impairment type, location and severity, and assigning the swimmer to one of ten sport classes for freestyle, backstroke, and butterfly (S1–S10), and nine classes for breaststroke (SB1–SB9), with lower class numbers indicating greater activity limitation [16].

Swimmers with central motor and neuromuscular impairment (CMNI) are particularly challenging to classify objectively as the impact of impairment type and severity on the determinants of swimming performance has not been well researched. CMNI encompasses impairment types that affect movement coordination, including hypertonia, ataxia, athetosis, and impaired muscle power, often resulting from health conditions such as spinal cord injury, cerebral palsy, or other neuromuscular disorders [18]. Unlike anthropometric impairments (e.g., limb deficiency), CMNI swimmers may exhibit awkward, extraneous, uneven, or inaccurate movements [19] which can disrupt the rhythmic and coordinated techniques required for front crawl [20], particularly in those with impaired lower limbs or paralysis on one side of the body [18, 21]. As their activity limitations vary considerably based on the type, severity, and location of central nervous system pathology [22], CMNI swimmers may be expected to present a more diverse range of body roll patterns compared to competitive swimmers without an impairment. How CMNI swimmers create their movements in the water, including body roll, will be influenced by the level of function they possess in the upper limbs, trunk, and lower limbs. They may not be able to employ effectively the mechanisms used by non-disabled swimmers to generate body roll. For example, an upper limb impairment may reduce the capacity to generate hydrodynamic torque to drive torso rotation, while a lower limb impairment may diminish the internal reaction torques available to control body roll.

Professionals involved in teaching, coaching or classifying swimmers with CMNI, and the athletes themselves, may benefit from new knowledge of how these impairments influence body roll kinematics and, consequently, front crawl swimming. This study aims to: (i) examine the effect of CMNI on body roll kinematics, (ii) evaluate the strength of association between body roll kinematics, sport class and swimming variables (swimming speed, stroke frequency, stroke length), and (iii) establish whether levels of upper-limb and lower-limb function influence body roll kinematics within the CMNI group. It is hypothesised that due to reduced function in their limbs or torso: (i) CMNI swimmers’ body roll kinematics deviate from non-disabled competitive swimmers’, and (ii) the association between body roll kinematics and swimming variables differs between CMNI and non-disabled competitive swimmers. It is also hypothesised that (iii) CMNI swimmers’ body roll range is positively associated with sport class, as those in lower sport classes (more severely impaired) may be less able to generate the required torques to drive body roll than those in higher sport classes, and that (iv) body roll kinematics differ according to the level of upper-limb and lower-limb function, as swimmers with a more severe upper or lower limb impairment may adopt different body roll strategies to those with less severe impairment of the same limbs, to manage the different constraints imposed by their physical impairment.

## Methods

### Participants

Twenty-seven competitive Para swimmers (17 males; 10 females) were recruited from four national squads using purposeful, convenience sampling [23]. All were nationally or internationally classified with an eligible CMNI impairment, injury-free and in full-training at the time of testing. Two participants performed front crawl with a six-beat flutter kick, the others used only one or neither lower limb due to their impairments. The mean best long course time of CMNI swimmers was 48.2 ± 16.0 s for 50 m freestyle. Participant details are presented in Table 1. Thirteen non-disabled competitive swimmers (11 males; 2 females; 22 ± 3 years; 185.8 ± 6.7 cm; 82.8 ± 8.2 kg) were also included as a comparison group. Their mean best long course time was 50.5 ± 2.5 s for 100 m freestyle. The study was granted ethical approval by Manchester Metropolitan University Ethics Committee and written informed consent was obtained from all participants.

**Table 1:** Characteristics of Para swimmers and their allocated upper-limb and lower-limb function subgroups

Sport class	Sex	Age (years)	Height (cm)	Mass (kg)	PB 50 m (s)	Health condition	Function subgroups	
							Upper-limb	Lower-limb
S9	M	28.0	170.1	85.7	38.9	Arnold-Chiari malformation	UL_mild_	LL_bilateral_
S8	M	22.5	176.6	65.2	42.9	CP diplegia	UL_mild_	LL_unilateral_
S8	F	19.1	142.2	45.5	37.5	Incomplete L4 SCI	UL_mild_	LL_unilateral_
S8	M	24.7	175.0	70.4	29.0	Muscle dystrophy	UL_mild_	LL_bilateral_
S8	M	19.2	171.0	65.0	28.7	Spina bifida L4/5	UL_mild_	LL_without kick_
S8	M	37.0	179.1	91.0	50.6	Incomplete C6 SCI	UL_moderate_	LL_unilateral_
S8	M	26.1	177.0	65.0	31.4	CP diplegia	UL_moderate_	LL_without kick_
S7	F	25.8	161.0	63.0	58.4	CP diplegia	UL_severe_	LL_unilateral_
S7	F	24.1	170.0	62.1	39.3	Strumpell-Lorrain Syndrome	UL_mild_	LL_without kick_
S6	M	20.4	146.0	56.3	59.2	Diastematomyelia	UL_severe_	LL_without kick_
S6	M	21.6	173.0	50.2	40.1	Complete spina bifida L2/3	UL_moderate_	LL_without kick_
S6	F	17.8	160.5	58.5	45.0	CP quadriplegia	UL_mild_	LL_without kick_
S6	M	16.9	157.7	53.0	31.0	Incomplete T7 SCI	UL_moderate_	LL_without kick_
S6	M	24.2	163.8	70.0	32.9	CP spastic diplegia	UL_moderate_	LL_without kick_
S6	M	29.6	176.6	74.8	41.3	CP diplegia	UL_mild_	LL_without kick_
S6	M	22.0	170.5	65.3	58.6	CP diplegia	UL_moderate_	LL_without kick_
S6	F	23.0	163.0	52.0	49.9	CP spastic diplegia	UL_moderate_	LL_without kick_
S5	F	22.8	160.0	89.0	82.4	Arthrogryposis	UL_severe_	LL_unilateral_
S5	F	24.4	153.1	50.0	52.8	Complete T10 SCI	UL_severe_	LL_without kick_
S5	M	24.2	162.3	64.0	45.2	CP spastic diplegia	UL_moderate_	LL_without kick_
S5	M	24.3	169.4	66.9	36.7	CP spastic hemiplegia	UL_severe_	LL_unilateral_
S4	M	38.6	174.0	74.0	50.6	Complete T8 SCI	UL_severe_	LL_without kick_
S4	F	31.3	151.0	55.0	42.4	Hereditary spastic paraplegia	UL_moderate_	LL_without kick_
S4	F	19.9	159.5	54.0	38.6	CP spastic diplegia	UL_mild_	LL_without kick_
S4	M	28.3	176.0	75.0	89.5*	CP quadriplegia	UL_severe_	LL_without kick_
S3	F	35.8	164.2	68.4	73.2*	Incomplete C5 SCI	UL_severe_	LL_without kick_
S2	M	29.3	159.6	53.0	76.5	Polyneuropathy	UL_severe_	LL_without kick_

S = Sport Class, PB = personal best, M = male, F = female, CP = cerebral palsy, SCI = spinal cord injury, UL = upper-limb, LL = lower-limb. ^*^Based on time trial

* Personal best 50 m front crawl record was calculated based on the swimmer's time trial

### Data Capture and Processing

Data collection took place at six sites in 25 and 50 m indoor pools with depths ranging from 1.8 to 2.0 m. After a warmup with self-selected volume and intensity, participants performed two 25 m front crawl trials at 100–200 m race pace from a push start with at least 3 min’ rest between trials. A floating frame containing 108 control points was used to calibrate a performance volume (1.50 m (*x*) $\times$ 3.75–6.00 m (*y*) $\times$ 1.80 m (*z*)) and establish a global right-handed Cartesian coordinate system with orthogonal axes in the right lateral (*x*), swimming (*y*), and vertical (*z*) directions. Swimmers were asked to hold their breath as they swam through the performance volume to control for the effect of the breathing action [7]. Each trial was recorded below water via four full HD Ethernet cameras (Mako G-223B, Allied Vision Technologies GmbH, Germany) in waterproof housings (Nautilus IP68, Autovimation GmbH, Germany) mounted on tripods approximately 1 m below the surface, and above water using four additional full HD Ethernet cameras or full HD camcorders (Sony HDR-CX700, Sony Corporation, Japan). Video data were captured to a PC hard drive using commercial software (Gecko GigE video recorder v1.9.4, Vision Experts Ltd, England). Camera positions and orientations were similar to those previously described [24].

Seventeen anatomical landmarks, defining a 14-segment model of the body, were marked to aid digitisation. The estimated joint centres/segment endpoints were then digitised manually for each video frame (50 Hz) using SIMI Motion 9.2.2 (SIMI Reality Motion Systems GmbH, Unterschleißheim, Germany). Real-world 3D coordinates were obtained using a DLT algorithm [25] and then smoothed using a 2nd order Butterworth low pass filter with a cut-off of 6 Hz [24]. Whole-body mass centre location was estimated using inertia data from de Leva [26].

### Data Analysis

Associations between sport class and all discrete body roll variables initially were explored using Kendall’s tau coefficients but none were significant. Since body roll is partly driven by hydrodynamic forces produced by the upper and lower limbs [2], we decided to explore how upper and lower limbs influence body roll kinematics separately. By regrouping the CMNI cohort into three upper-limb function subgroups and three lower-limb function subgroups, we sought to establish whether the location and level of function have an impact on body roll kinematics.

#### Defining Upper-Limb and Lower-Limb Function Subgroups

Upper-limb function was defined by calculating each swimmer’s Froude efficiency (*η*_F_), an established measure of how effectively upper limbs contribute to propulsion, using the method proposed by Figueiredo et al. [27] (for a detailed review of swimming efficiency refer to Zamparo et al. [28]). Upper-limb (UL) function subgroups were established as follows: (i) UL_severe_: *η*_F_ 0.16–0.27, (ii) UL_moderate_: *η*_F_ 0.28–0.32, and (iii) UL_mild_: *η*_F_ 0.33–0.42. Foot vertical speed was used to define leg kick activity. In non-disabled and CMNI swimmers with bilateral kicks, values ranged from 2–3 m·s^−1^ with symmetrical downbeat and upbeat phases (differences < 25%). A lower-limb was considered passive when the foot speed curve was irregular and sporadic, speeds were below 1 m·s^−1^ and asymmetry exceeded 50%. Lower-limb (LL) function subgroups were defined by the number of lower limbs actively used during the trials, as follows: (i) LL_without kick_: no lower limb, (ii) LL_unilateral_: one lower limb, and (iii) LL_bilateral_: both lower limbs.

#### Dependent Variables

A complete cycle of the upper limbs, defined as the period from one hand entry into the water to the next same hand entry, was analysed. The following variables were obtained: *Swimming speed* (m·s^−1^) – mean horizontal (y-axis) whole-body mass centre velocity for the cycle. *Stroke frequency* (stroke∙min^−1^) – reciprocal of the cycle time multiplied by 60. *Stroke length* (m) – displacement of mass centre in the y-direction during the cycle. Shoulder and hip roll angles were the angles created by the projections of the vectors linking each bilateral joint pair (glenohumeral and hip) onto the x–z plane, with the horizontal. The following body roll kinematic variables were calculated for the shoulders and the hips within the cycle: (i) *roll range* (°) – sum of the absolute values of the maximum and minimum roll angles; (ii) *roll asymmetry* (°) – the difference between the absolute values of the maximum and minimum roll angles; (iii) *right/left side maximum torso twist* (°) – the maximum difference between shoulder roll and hip roll on each side; (iv) *range of torso twist* (°) – sum of the maximum magnitude of torso twist from the left and right sides; (v) *roll phase lag* (%) – time difference between cessation of shoulder roll and cessation of hip roll, expressed as a percentage of the cycle time. Roll phase lag defines how far the shoulder roll lags behind the hip roll, with positive values denoting hip rotation ending before shoulder rotation ends, and negative values denoting shoulder rotation ending before hip rotation ends.

### Statistical Analysis

IBM SPSS Statistics 28 (IBM Corp., Armonk, NY, USA) and MATLAB R2024a (The Mathworks Inc., Natick, MA, USA) were used to analyse discrete and continuous data, respectively. The threshold for statistical significance was set at *p* < 0.05. Female and male data were pooled for all analysis as body roll variables and Froude efficiency have not been shown to be sex dependent. Nonparametric tests were used where variables were not normally distributed.

To test for differences in discrete body roll variables between the CMNI and non-disabled groups, Mann–Whitney U tests were performed and Rank-Biserial Correlations (*r*_*rb*_) were calculated as a measure of effect size. To compare discrete body roll variables between the three upper-limb function subgroups, a one-way MANOVA was used. Multiple comparisons were made using Bonferroni corrected post hoc pairwise comparisons and partial eta squared ($\eta_{p}^{2}$) was calculated as a measure of effect size. As LL_bilateral_ subgroup contained only two swimmers it was excluded from the main analysis and three-sigma limits (99.7% confidence interval) [29] was used to compare it to the other two lower-limb function subgroups. LL_without kick_ and LL_unilateral_ subgroups were compared using Kruskal–Wallis H tests. To assess the strength of association between swimming variables and body roll variables, Spearman’s rank correlation coefficients were defined as trivial (< 0.1), weak (0.1–0.3), moderate (0.3–0.5), large (0.5–0.7), very large (0.7–0.9), and extremely large (> 0.9) [30].

Statistical non-parametric mapping (SnPM) was used to compare body roll kinematics over a complete cycle between: (i) the CMNI and non-disabled groups, (ii) LL_without kick_ and LL_unilateral_ subgroups using two-tailed non-parametric t-tests, and (iii) the three UL subgroups using non-parametric ANOVAs. SnPM analyses were conducted using the SPM1D (v.M.04) code in MATLAB R2024a (The Mathworks Inc., Natick, M.A., USA) to calculate the magnitude of differences, at each individual time node, with a critical threshold of $\alpha$ = 0.05. A supra-threshold cluster was defined when multiple adjacent points of the SnPM trajectory cross the critical threshold.

#### Reliability

Repeatability of the body roll curve data was assessed following the procedures of Sanders et al. [6]. One upper-limb cycle was digitised five times by the same operator from each camera view to generate five independent 3D data sets from which the body roll variables were calculated. For each variable, the standard deviation was calculated at every time point in the cycle with absolute measurement error (*ME*_ABS_) defined as the mean of the standard deviations; relative error (*ME*_REL_) was the absolute error expressed as a percentage of the total range of the variable. Coefficient of multiple determination (*CMD*) was used to evaluate the reliability of the angle-time series data. Coefficients of multiple determination ranged from 0.95 to 1.00 indicating the body roll curves had very good repeatability (*CMD*: hip roll *r* = 1.00, shoulder roll *r* = 0.99, torso twist *r* = 0.95). Shoulder and hip roll errors were acceptably small (Hip roll: *ME*_ABS_ = 0.68°, *ME*_REL_ = 0.77%; shoulder roll: *ME*_ABS_ = 1.62°, *ME*_REL_ = 2.42%) as was the torso twist absolute error (*ME*_ABS_ = 1.86°). The torso twist relative error (*ME*_REL_ = 5.72%) was higher due to the relatively low range of torso twist and a propagation of errors from the two variables used to compute the twist angle.

## Results

### Comparison Between Non-disabled and CMNI Groups

Table 2 presents discrete body roll variables for the non-disabled group, the overall CMNI swimmer group, and for the upper-limb and lower-limb function subgroups. Compared to non-disabled swimmers, CMNI swimmers exhibited significantly lower swimming speed (*p* < 0.001, *r*_*rb*_ = 0.994), stroke length (*p* < 0.001, *r*_*rb*_ = 0.974), range of shoulder roll (*p* = 0.006, *r*_*rb*_ = 0.538) and torso twist (*p* = 0.045, *r*_*rb*_ = 0.396) but significantly greater range of hip roll (*p* = 0.045, *r*_*rb*_ = − 0.396). Stroke frequency, shoulder roll asymmetry, hip roll asymmetry, and maximum torso twist and roll phase lag on both left and right sides, did not differ between swimmers with and without an impairment.

**Table 2:** Front crawl body roll kinematics for thirteen non-disabled swimmers and twenty-seven swimmers with central motor and neuromuscular impairments, presented as a pooled group, three levels of upper-limb function subgroups and three levels of lower-limb function subgroups

	Non-disabled (*n* = 13)	CMNI (*n* = 27)	Upper-limb function subgroups (*n* = 27)			Lower-limb function subgroups (*n* = 27)		
			UL_severe_ (*n* = 9)	UL_moderate_ (*n* = 9)	UL_mild_ (*n* = 9)	LL_without kick_ (*n* = 19)	LL_unilateral_ (*n* = 6)	LL_bilateral_ (*n* = 2)
Mean swimming speed (m∙s^−1^)	1.72 ± 0.12	1.03 ± 0.30^a^	0.72 ± 0.23^cd^	1.18 ± 0.24^b^	1.18 ± 0.16^b^	1.02 ± 0.31	0.96 ± 0.29	1.20/1.44
Stroke frequency (stroke∙min^−1^)	43.8 ± 6.9	41.8 ± 9.2	36.1 ± 9.5^c^	48.2 ± 8.7^b^	41.1 ± 5.0	42.1 ± 10.6	40.7 ± 5.1	44.8/40.5
Stroke length (m)	2.40 ± 0.28	1.47 ± 0.32^a^	1.21 ± 0.30^d^	1.47 ± 0.20	1.73 ± 0.22^b^	1.45 ± 0.28	1.42 ± 0.40	1.61/2.13
Shoulder roll range (°)	104.1 ± 10.5	88.1 ± 21.3^a^	101.7 ± 25.7	82.1 ± 15.9	80.4 ± 15.6	83.1 ± 18.2	105.0 ± 26.7	85.2/82.5
Shoulder roll asymmetry (°)	9.8 ± 9.4	9.7 ± 6.8	9.4 ± 6.3	9.5 ± 8.9	10.2 ± 5.7	10.2 ± 7.6	6.8 ± 3.8	14.2/12.8
Hip roll range (°)	61.8 ± 9.6	74.9 ± 29.4^a^	79.6 ± 27.1	60.0 ± 31.0	85.2 ± 26.6	71.1 ± 32.1	82.8 ± 17.4	61.7/114.0
Hip roll asymmetry (°)	5.2 ± 5.5	9.7 ± 7.9	11.3 ± 10.5	8.8 ± 7.0	9.0 ± 6.2	9.3 ± 9.1	10.5 ± 3.9	6.9/15.8
Range of torso twist (°)	57.7 ± 13.4	48.1 ± 22.1^a^	49.5 ± 24.7	45.2 ± 19.6	44.2 ± 22.7	48.7 ± 21.6	49.7 ± 28.4	33.8/42.7
Left side max torso twist (°)	30.6 ± 9.5	24.3 ± 15.0	23.6 ± 16.8	24.1 ± 14.5	22.6 ± 15.3	24.7 ± 14.7	25.4 ± 19.3	15.4/19.6
Right side max torso twist (°)	27.1 ± 7.5	23.8 ± 9.4	25.9 ± 9.6	21.1 ± 8.2	21.6 ± 9.3	24.0 ± 9.9	24.3 ± 10.0	18.4/23.1
Roll phase lag (%)								
Greater shoulder roll side	4.6 ± 8.1	4.0 ± 9.5	5.4 ± 9.4	0.7 ± 8.7	5.9 ± 10.6	3.4 ± 10.6	8.0 ± 5.5	0/− 3.9
Lesser shoulder roll side	5.4 ± 9.8	1.0 ± 11.2	5.8 ± 11.0	− 5.2 ± 11.9	2.4 ± 8.4	− 0.5 ± 11.6	7.2 ± 9.9	− 1.5/− 5.2

Data are reported as mean ± SD. *Abbreviation:* CMNI = central motor and neuromuscular impairment; UL = upper-limb function subgroups; LL = lower-limb function subgroups. ^a^Denotes a significant difference between CMNI and non-disabled group; ^b^Denotes a significant difference from UL_severe_ subgroup; ^c^Denotes a significant difference from UL_moderate_ subgroup; ^d^Denotes a significant difference from UL_mild_ subgroup

**Fig. 1:**
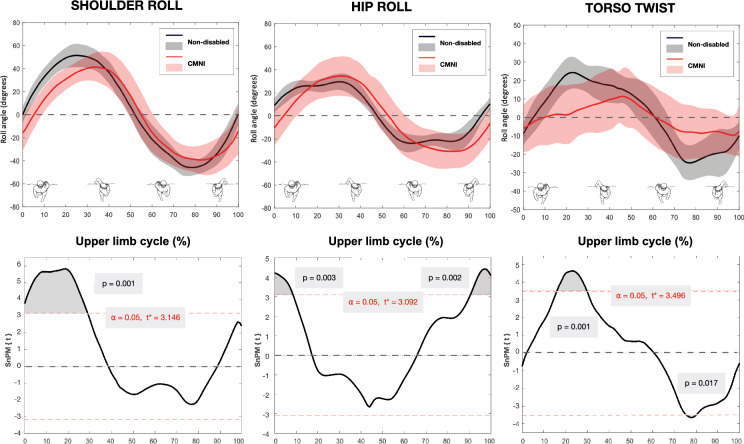
Shoulder roll, hip roll and torso twist in non-disabled swimmers and swimmers with central motor and neuromuscular impairments (CMNI) during an upper limb cycle (*top row*). Black solid lines (*grey shading*) and red solid lines (*red shading*) represent the mean (± 1 SD) for the non-disabled group and the CMNI group, respectively. Curves begin (0%) at hand entry and end (100%) at the next same hand entry. Corresponding statistical non-parametric maps (*bottom row*) indicate where data passed the critical threshold (*grey shaded area*)

Comparisons of shoulder roll, hip roll and torso twist between CMNI and non-disabled swimmers for a normalised upper limb cycle are shown in Fig. 1. CMNI swimmers exhibited less shoulder roll than non-disabled swimmers at one supra threshold cluster (0–28%, *p* = 0.001). Hip roll was less at one supra threshold cluster (0–10%, *p* = 0.003) and greater at one supra threshold cluster (91–100%, *p* = 0.002) in the CMNI group than non-disabled group. Greater torso twist was observed in the non-disabled group than the CMNI group at two occasions within the cycle (15–32%, *p* = 0.001; 75–81%, *p* = 0.017). The non-disabled swimmers demonstrated considerably less variable shoulder roll, hip roll and torso twist compared to the CMNI group, as evidenced by the standard deviations (grey shading) in Fig. 1.

For both CMNI and non-disabled groups, range of hip roll had strong negative associations with stroke frequency (*r*_s___CMNI_ = − 0.599, *p* = 0.001; *r*_s___non-disabled_ = − 0.677, *p* = 0.011) and stroke length (*r*_s___CMNI_ = 0.541, *p* = 0.007; *r*_s___non-disabled_ = 0.716, *p* = 0.006). In contrast, stroke frequency and stroke length were not correlated with range of shoulder roll for either group (*p* > 0.05). Range of shoulder roll showed moderate positive associations with range of hip roll (*r*_s_ = 0.478, *p* = 0.014) and torso twist (*r*_s_ = 0.397, *p* = 0.044) in CMNI swimmers; for the non-disabled group it was again associated with torso twist (*r*_s_ = 0.698, *p* = 0.010) but not with range of hip roll (*p* > 0.05). A strong positive association between range of hip roll and hip roll asymmetry was observed in the CMNI group only (*r*_s_ = 0.625, *p* < 0.001).

### Effect of Upper-Limb and Lower-Limb Function

No significant association was found between the CMNI swimmers’ sport class and any of the body roll variables. The mean swimming speed of CMNI swimmers with severe upper-limb function was lower compared to those with moderate and mild upper-limb functions (*p* < 0.001, $\eta_{p}^{2}$ = 0.53). No significant difference in mean speed was observed between UL_moderate_ and UL_mild_. The stroke length of UL_severe_ was lower than UL_mild_ (*p* < 0.001, $\eta_{p}^{2}$ = 0.47). No significant differences in stroke length were found between other upper-limb subgroup pairs. Stroke frequency of UL_severe_ was lower than UL_moderate_ (*p* = 0.011, $\eta_{p}^{2}$ = 0.31). Stroke frequencies did not differ significantly between other upper-limb subgroup pairs. Comparisons between the lower-limb function subgroups revealed no significant differences in any of the variables in Table 2.

**Fig. 2:**
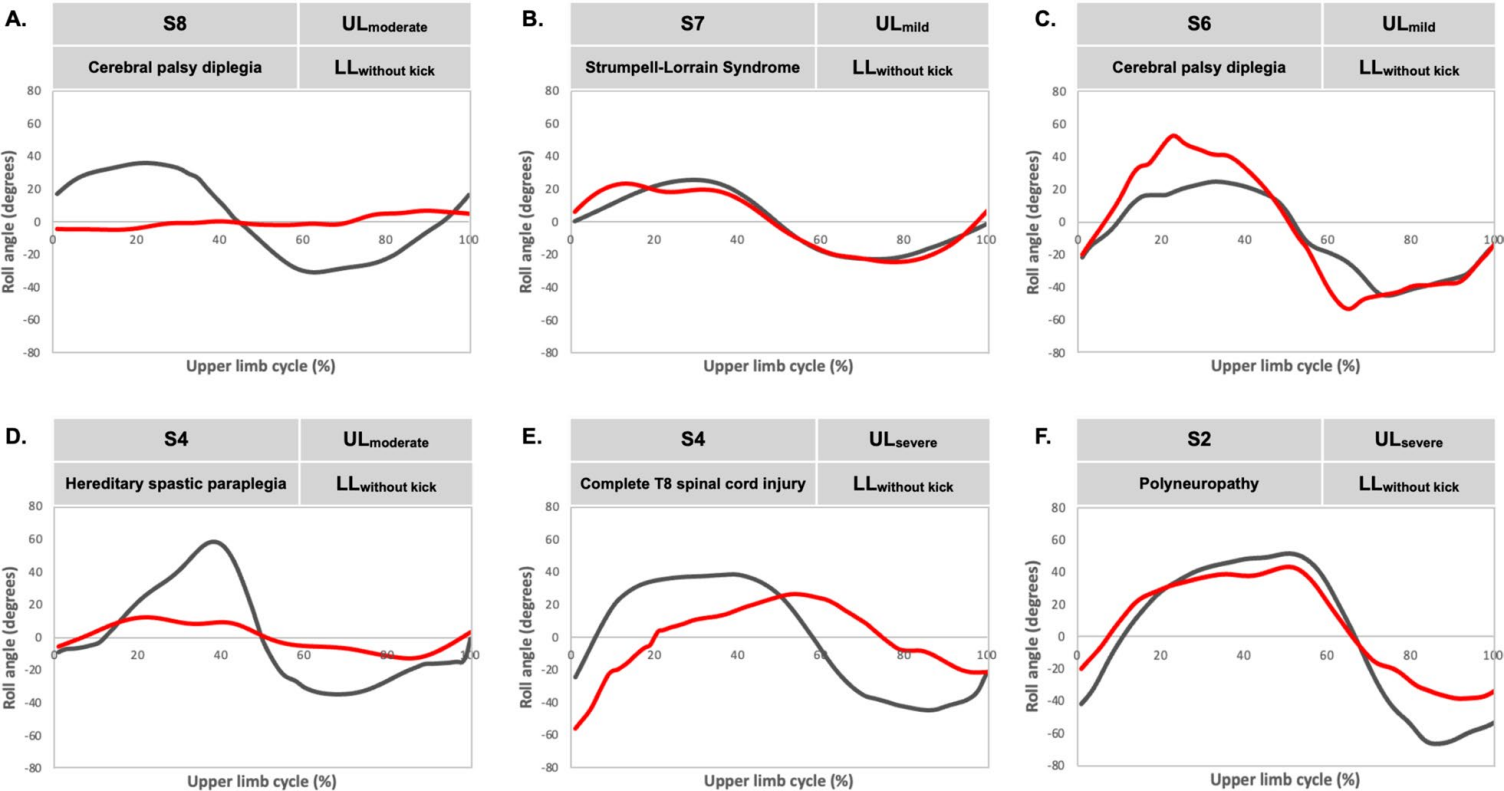
Examples of shoulder roll (black solid lines) and hip roll (red solid lines) during an upper limb cycle in swimmers with central motor and neuromuscular impairments. Each example includes a Para swimmer’s sport class (S), health condition, upper-limb (UL) and lower-limb (LL) function subgroups. Curves begin (0%) at hand entry and end (100%) at the next same hand entry

Figure 2 presents shoulder roll and hip roll curves for six CMNI swimmers to illustrate the extent to which some swimmers’ profiles deviated from the ensemble averages shown in Fig. 1. Swimmer A exhibited negligible hip roll throughout the cycle; swimmer B presented with minimal torso twist throughout the cycle; swimmer C’s hip roll exceeded his shoulder roll; swimmer D had ~ 20° difference between shoulder roll peak angles; swimmer E’s hip roll peak lagged ~ 25% of the cycle duration behind his shoulder roll peak; the hips and shoulders of swimmer F reached neutral (0°) at ~ 10% and ~ 70% of the cycle.

**Fig. 3:**
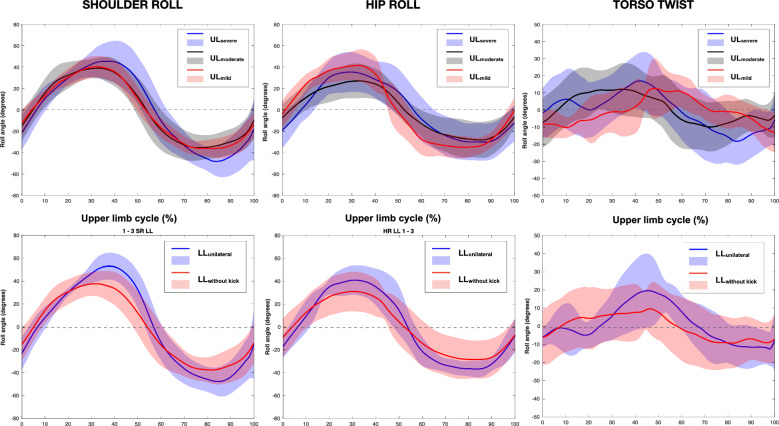
Shoulder roll, hip roll and torso twist during an upper limb cycle between three levels of upper-limb (UL) function subgroups (*top row*) and between two levels of lower-limb (LL) function subgroups (*bottom row*) in swimmers with central motor and neuromuscular impairments. The solid lines represent the mean of each subgroup and shaded areas represent one standard deviation. Curves begin (0%) at hand entry and end (100%) at the next same hand entry

Figure 3 presents time-series data for shoulder roll, hip roll and torso twist for the three upper-limb subgroups and two lower-limb subgroups. SnPM revealed no differences in these variables across the cycle between any subgroups. Range of hip roll, shoulder roll asymmetry and hip roll asymmetry, torso twist, left and right maximum torso twist, roll phase lag (*p* > 0.05) and range of shoulder roll (*p* = 0.056, ${\eta }_{p}^{2}$ = 0.21) did not differ between upper-limb function subgroups or between the lower-limb function subgroups.

## Discussion

### Comparison of CMNI to Non-disabled Swimmers

This study is the first to present a detailed analysis of front crawl body roll kinematics of competitive CMNI swimmers. Compared to competitive non-disabled swimmers, CMNI swimmers exhibited lower shoulder roll range and torso twist but greater hip roll range. Their swimming speed and stroke length were also lower. Hip roll range had moderate to strong associations with stroke frequency and stroke length for both groups. A moderate association between shoulder roll range and hip roll range was found in CMNI swimmers but not in the non-disabled group. Hypothesis i, that CMNI swimmers’ body roll kinematics deviate from non-disabled competitive swimmers’, was thus accepted. Hypothesis ii, that the association between body roll kinematics and swimming variables differs between CMNI and non-disabled swimmers, was rejected as both groups showed similar associations for most of the comparisons made.

CMNI swimmers used similar stroke frequencies to the non-disabled group yet exhibited significantly less shoulder roll, indicating a reduced roll angular velocity. Given that swimmers require increased torque to produce a greater shoulder roll velocity [13], this finding may indicate that swimmers with CMNI are less effective than non-disabled competitive swimmers at creating non-propulsive hydrodynamic forces with their upper limbs to generate the torque to drive body roll. The impaired upper-limb function of the CMNI swimmers also limits their capacity to generate propulsive forces, as evidenced by their swimming speeds and stroke lengths being on average 40% and 39% lower, respectively, than those of the non-disabled swimmers. Upper-limb deformities that would have diminished hydrodynamic force generation included thumb-in-palm, swan neck and finger or wrist flexor deformity [31]. Another potential factor influencing CMNI swimmer body roll and performance will be their body alignment in the water. Due to the absence of leg kick, CMNI swimmers’ bodies were more inclined than those of the non-disabled swimmers and some of the most impaired participants swam with their lower-limb joints permanently flexed due to muscle contractures [32]. These different body orientations will likely increase the swimmer’s moment of inertia about the body roll axis and create an associated increase in hydrodynamic torque resisting body roll. The adverse consequences of the CMNI swimmers rolling their shoulders less than the non-disabled swimmers may include an increase in form drag, due to a greater projected frontal area presented to the water [11], and a decrease in stroke length due to a reduced glide or stretch of the arm in front of the head [12]. If CMNI swimmers attempted to roll their shoulders to the same extent and as fast as the non-disabled group, they would likely encounter difficulties in controlling their rolling action due to a partial or full impairment of lower limbs [2]. Greater shoulder roll may not be desirable or indeed optimal for performance of some CMNI swimmers who may deliberately limit their shoulder roll to prioritise stability of the body position in the water and also to direct more of the hydrodynamic force, created by their upper limbs, in the swimming direction for propulsive purposes.

The majority of CMNI swimmers performed front crawl without an active kicking motion thereby reducing their options for generating internal and external torques to initiate and control their body roll [2]. With a partial or full impairment of the kicking motion the vertical hydrodynamic forces on the lower limbs, and the internal reaction torques acting on the lower trunk, would be diminished allowing the hips to continue to roll passively further than they would have if a kicking motion had been present. This may explain why CMNI swimmers had greater hip roll range than their non-disabled counterparts and is supported by previous research that showed a strong and effective leg kick contributes to a reduction in hip roll [13, 33]. The positive association between shoulder roll range and hip roll range in the CMNI group, but not in the non-disabled swimmers may indicate different sources of torque driving the hip roll. The significantly reduced torso twist displayed by the CMNI group was due to them using lower shoulder roll and greater hip roll than the non-disabled group. As increased torso twist has been associated with an increase in swimming speed [13, 34], differences in swimming speed between the CMNI and non-disabled groups may account for some of the difference in torso twist, but it seems likely that the effects of impaired limb function, such as a weak kicking motion, are more responsible for the reduced torso twist. Due to cerebral palsy, spinal cord injury and neuromuscular disorders, our CMNI swimmers may have less control of torso muscles [35, 36] than non-disabled swimmers. How this influenced their torso twist or other body roll variables remains unclear. In non-disabled front crawl swimming the main function of the torso muscles appears to be to maintain stability and control posture, rather than to create torso twist [37]. Thus reduced function in the torso muscles may present CMNI swimmers with difficulties in sustaining a stable body position [35] rather than in generating torso twist, the latter being more a consequence of upper-limb and lower-limb actions.

No difference was found in the mean roll phase lag between swimmers with and without an impairment. Both groups recorded a positive phase lag indicating that, on average, the hips reached maximal rotation before the shoulders which corresponds with previous research findings [1]. The large variability in phase lag within both groups reflects the range of different shoulder-hip roll coordination strategies used by the swimmers, supporting the view that this variable is not associated directly with performance and that optimal roll patterns are specific to the individual [1]. While some CMNI swimmers displayed very atypical body roll profiles (Fig. 2), these patterns likely reflect the swimmers’ unique self-organising adaptation to the individual organism constraints imposed by their physical impairment [38] and should not necessarily be considered suboptimal or poor technique [39, 40]. Asymmetries in shoulder and hip roll for the CMNI swimmers were very similar to those of the non-disabled group and to values reported previously for non-disabled competitive swimmers [1]. Asymmetrical roll occurs for various reasons including breathing asymmetry [7], laterality, bilateral strength asymmetry, and imbalance in hydrodynamic forces from dominant and non-dominant limbs [1]. These factors may be amplified by the presence of an upper-limb or lower limb-impairment, particularly where that impairment is unilateral [3].

### Effect of Upper-Limb and Lower-Limb Function

No association was found between sport class and any body roll variable, and none of the body roll variables differed significantly between upper-limb and lower-limb function subgroups. Hence, hypothesis iii, that CMNI swimmers’ body roll range is positively associated with sport class, and hypothesis iv, CMNI swimmers’ body roll kinematics differ according to their level of upper-limb and lower-limb function, were both rejected.

As each Para swimming sport class comprises swimmers with various impairment types and activity limitations, swimmers within the same sport class often displayed quite different body roll patterns, compare for example Fig. 2D and E. This within-class diversity explains the absence of an association between sport class and any body roll variable, and provided a rationale for examining the effects of upper-limb and lower-limb function on body roll kinematics separately. Swimming speed, stroke length and stroke frequency all differed between the upper-limb subgroups as the groups were formed on the basis of their Froude efficiencies. Body roll kinematics were not different between upper-limb subgroups but there was a trend toward a lower shoulder roll in swimmers with higher Froude efficiencies and swimming speeds. This finding is consistent with previous research [1, 5]. The UL_severe_ subgroup achieved a similar shoulder roll range to the non-disabled swimmers but they rotated at a reduced rate due to their lower stroke frequency.

Body roll kinematics did not differ significantly between the lower-limb subgroups. Although the greatest range of hip roll was exhibited by a LL_without kick_ swimmer, the hip roll in this subgroup ranged from 12° to 132°. This high variability may be attributable to their diverse lower-limb orientations in the water. For instance, six of the swimmers, whose lower limbs remained flexed due to severe muscle contractures, had difficulties rotating their bodies. Their large frontal area could lead to more active drag during front crawl [1] as well as increase their moment of inertia about the body roll axis. The remaining LL_without kick_ swimmers could fully extend their lower limbs and achieve a more streamlined body position. Some of these had no control of their hip or torso musculature such that one swimmer’s hips rolled 36° more than their shoulders; others may have deliberately tried to minimise their hip roll to prevent their lower limbs from oscillating laterally. Given that bilateral six-beat kicking contributes to non-disabled front crawl body roll [33], the LL_unilateral_ subgroup was expected to exhibit the greatest hip roll asymmetry. Despite only having one active lower limb, these swimmers were able to utilise either buoyancy or hip extensor muscles to lift their dysfunctional limb in the water and compensate for the imbalanced torque caused by unilateral kicking. The greatest hip roll asymmetry was apparent in those LL_without kick_ swimmers with flexed lower limbs due to severe muscle contractures. They typically swam with marked bilateral asymmetry in their lower limb orientations, for example crossed legs, which would have contributed to their asymmetrical hip roll. That no differences were found between upper-limb subgroups or between lower-limb subgroups highlights that shoulder roll and hip roll are not determined solely by upper or lower limb actions but may also be achieved by torso twist [14].

The International Paralympic Committee has instructed the development of new evidence-based classification systems for each Para sport [41]. Key steps towards achieving this in Para swimming are to establish the impact of impairment type and severity on the determinants of performance, and to develop more valid land-based measures of impairment that only assess body structures that impact performance, in body positions relevant to performance [18]. This study is the first to establish how central motor and neuromuscular impairments affects body roll, a critical determinant of front crawl technique, and performance. The new knowledge generated can be used to inform and improve both the land-based physical assessment (bench test) and the water-based technical assessment currently used to classify CMNI swimmers.

Bench tests assess the level of impairment and activity limitation of an athlete and the extent to which the activity limitation impacts the athlete’s sporting performance [16]. World Para Swimming’s bench test for Passive Functional Range of Movement deems Para swimmers to have no restriction in trunk rotation (zero points) if their torso twist reaches 50° or more to each side in a seated test. Conversely, a maximum torso twist in the range 12–25° is considered ‘restricted’ by 50–75% and receives 3 points for each side. Our in-water measures show that Para swimmers and non-disabled swimmers reach maximum torso twist angles of ~ 24–30° which is 40–50% below the bench-test threshold used to designate no restriction for this movement. This indicates that swimmers may currently be receiving unwarranted points for trunk activity limitation, as the torso twist required to perform front crawl is far lower than the functional range of movement angle used in the bench test. This finding highlights the need to reevaluate the current range of motion thresholds used to allocate points to other joint motions during the physical assessment to ensure they are fit for purpose.

In the technical assessment, classifiers assess the impact of the athlete’s impairment on the different swimming strokes. This includes the ability to ‘control their body…with particular attention to body streamline, body roll, trunk control and leg kick for balance…’ [16]. Trunk movement is scored from 0 points for ‘no trunk control– no balance/stability’ up to 5 points for ‘full trunk control – normal balance/stability’ with the classifier required to interpret these descriptors. We propose that this study’s findings could inform the development of more detailed, practical guidelines for evaluating trunk movement in the technical assessment. Classifiers could be directed to the key variables to be observed (shoulder roll, hip roll, torso twist, phase lag and asymmetry) and provided with benchmark data enabling them to assess the extent to which the observed athlete’s trunk movements deviate from those of non-disabled competitive swimmers.

This study has limitations and there are areas where further work is necessary. Our participant cohort comprised swimmers with cerebral palsy, spinal cord injury and other neuromuscular disorders, so the results are not generalisable to Para swimmers with a specific health condition. Participants were tested in a non-fatigued state at a single speed and our analysis was limited to a single upper limb cycle, which was assumed representative of the swimmer’s typical technique. The effects of swimming speed, fatigue and inter-cycle variability on body roll have not therefore been addressed in this study. Finally, the small sample size of two of the lower-limb function subgroups precluded the use of statistical analysis to assess differences between subgroups.

## Conclusions

This study has quantified body roll kinematics in swimmers with CMNI and shown that they use less shoulder roll and torso twist, but more hip roll, than non-disabled swimmers, likely due to reduced upper-limb function, poor torso control, a partial or full impairment of the lower limbs, or a combination of all. Body roll characteristics of CMNI swimmers with severe upper-limb function do not differ significantly from those of swimmers with moderate or mild upper-limb function, and the number of lower limbs actively kicking does not have a clear effect on body roll kinematics. CMNI swimmers present greater variation in their body roll strategies compared to non-disabled swimmers reflecting the diverse range of constraints imposed by their physical impairments. During the physical assessment component of classification, Para swimmers may be receiving unwarranted points for restricted trunk rotation. Our study demonstrates that the torso twist required in front crawl is far lower than the range of movement threshold currently used to define activity limitation in the passive functional range of movement bench test.

## Data Availability

The data that support the findings of this study can be accessible from the corresponding author upon request.

## Abbreviations

CMNI: Central motor and neuromuscular impairment
*η*_F_: Froude efficiency
S: Sport class
UL: Upper-limb function subgroups
LL: Lower-limb function subgroups
